# Solvent-Based Separation of PA6 and PET Using Solubility Data from Differential Scanning Calorimetry

**DOI:** 10.1021/acs.iecr.5c04861

**Published:** 2026-02-27

**Authors:** Ruben Goldhahn, Ann-Joelle Minor, Liisa Rihko-Struckmann, Kai Sundmacher

**Affiliations:** † Department of Process Systems Engineering, 28307 Max Planck Institute for Dynamics of Complex Technical Systems, Sandtorstraße 1, Magdeburg 39106, Germany; ‡ Otto-von-Guericke University, Chair of Process Systems Engineering, Universitätsplatz 2, Magdeburg 39106, Germany

## Abstract

To minimize plastic pollution and prevent further extraction of fossil feedstock, the circular economy for polymers offers a promising solution. As an integral part of the circular economy to close material loops, solvent-based recycling enables the separation of mixed plastic waste into pure component streams without altering the polymer structure but requires reliable solubility data of polymers in solvents. We present a standardized differential scanning calorimetry (DSC) protocol to determine decrystallization and precipitation temperatures of polymers in various solvents and apply it to the challenging separation of polyamide 6 (PA6) and polyethylene terephthalate (PET). The DSC method enables quantitative solubility analysis even above solvent boiling points and shows good agreement with a visual polythermal cloud point method while offering higher objectivity and additional phase transition enthalpies. Temperature-dependent solubility data for nine solvents reveal that methanol, ethanol, propylene glycol, dimethyl sulfoxide, and water are selective solvents for PA6 over PET. A proof-of-concept PA6-PET separation using methanol yields recycled polymers with near-virgin properties under mild conditions (109 °C, ∼4 bar), demonstrating the process relevance of the obtained DSC data for solvent selection in low-energy, solvent-based recycling processes.

## Graphical abstract

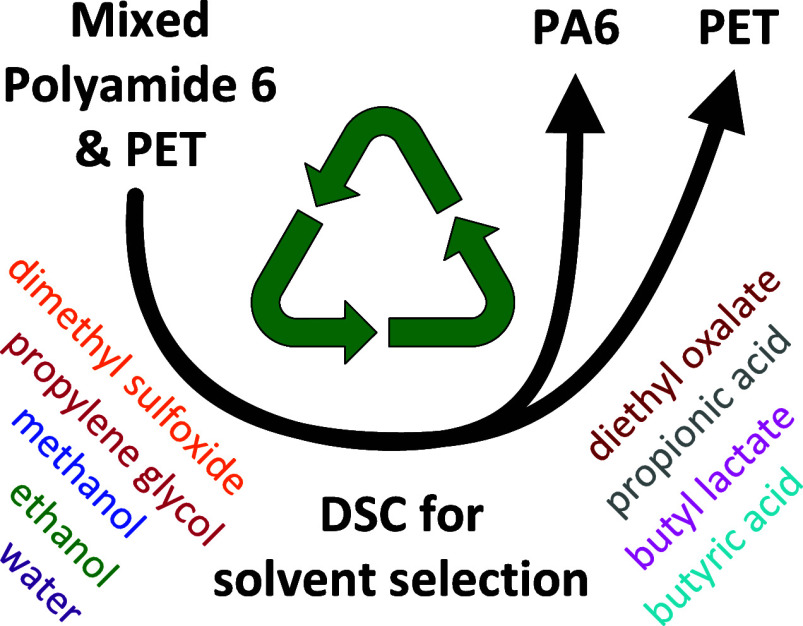

## 1 Introduction

In a circular economy, waste plastics must be recovered to serve as a raw material for the next cycle.^69,70^ A bottleneck for high-quality plastics recycling avoiding downcycling is the ineffectiveness of mechanical separation techniques.^1–3^ Especially, multilayered packaging or interwoven fibers in particular pose a challenge when it comes to separating them into their pure components using mechanical means alone.^4^ Polymers like polyamide 6 (PA6) which produce large amounts of waste and require huge amounts of energy in petroleum-based manufacturing^5^ are especially desirable to be recycled to mitigate pollution.

Solvent-based recycling, also known as the dissolution–precipitation process, offers the possibility to selectively dissolve one polymer from a mixture and thus enables successive separation of a mixed plastic waste stream into pure polymer streams. For PA6, solvent-based recycling (∼0.4–0.9 kg CO_2_/kg PA6) causes higher emissions than mechanical recycling (∼0.1 kg CO_2_/kg PA6)^6^ but lower than investigated chemical recycling processes (1.3–11.0 kg CO_2_/kg PA6).^68^ Thus, if mechanical recycling is infeasible, solvent-based recycling should be preferred over chemical recycling for avoiding a fossil-based production of virgin PA6 (4.5 kg CO_2_/kg PA6) with incineration or landfilling as end-of-life treatments.^6^

A process scheme for a solvent-based separation of waste PA6 (wPA) from waste PET (wPET) with temperature-induced precipitation is shown in Figure 1. First, wPA6 is selectively dissolved in the solvent at the dissolution temperature, *T*_diss_. Second, wPET is separated from the PA6 in solution at *T*_diss_ by mechanical solid–liquid separation (e.g., filter, centrifuge, decanter, hydrocyclone), and recycled PET (rPET) is obtained by evaporating any solvent residue at a drying temperature, *T*_dry_. Third, dissolved PA6 is cooled to the temperature *T*_prec_ for precipitating the polymer in the solvent. Fourth, the precipitated PA6 is mechanically separated from the solvent, the solvent is recycled, and the residual solvent is removed from PA6 at *T*_dry_ by solvent evaporation to obtain recycled PA6 (rPA). This solvent-based recycling process applying temperature-induced precipitation, also called the cooling method, has a lower energy demand and therefore improved economic feasibility compared to processes using an antisolvent or complete solvent evaporation to achieve polymer precipitation.^6,7–13^ Since lower energy inputs cause fewer emissions,^68^ this article focuses on solvents that enable temperature-induced precipitation of the polymer.

**Figure 1:**
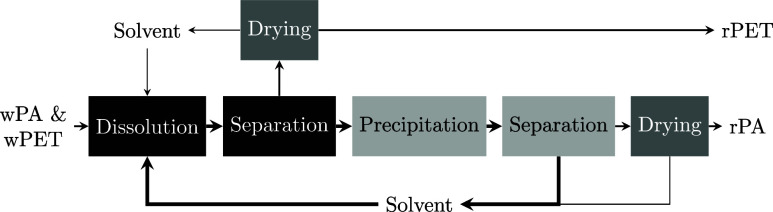
Process block flow diagram in which waste polyamide 6 (wPA) is selectively dissolved and separated from waste PET (wPET), with subsequent recovery of recycled PET (rPET), recycled polyamide (rPA), and a low-boiling or weak solvent for PA6. Boxes indicate processes, with darker colors corresponding to higher temperatures and arrow thickness indicating mass flow amounts.

PA6 is commonly one contributor in mixed plastic waste of multilayer packaging together with polyethylene (PE), polypropylene (PP), and polyethylene terephthalate (PET);^14–18^ carpets containing mostly PA, PP, styrene-butadiene rubber (SBR),^15,19–23^ and sometimes also PET;^24–27^ fishing nets composed of PA, PET, PP, and PE;^28^ textile fibers containing PA, PET, wool, and cotton;^26,29,30^ and the automotive shredder light fraction (SLF) that consists of PA, PP, PE, PET, polyurethane (PU), acrylonitrile-butadiene-styrene (ABS), and others.^31,32^ A few solvents selectively dissolve nonpolar PP^14,16,23,33^ or nonpolar PE^14,33,34^ from polar PA. However, finding a selective solvent for PA or PET that does not dissolve the other polymer remains challenging^11,13,16,35^ since both polymers contain polar functional groups (amide and ester bonds).

Many computational screening studies have been carried out to identify solvents to selectively dissolve polymers from a mixture of plastics, but only a few of these included an experimental validation.^4,7,36–43^ However, while predictions of computational models provide a powerful first filter, experimental confirmation is needed to capture practical effects. Additionally, isothermal gravimetric methods may be confounded by solvent retention in the polymer, and several theoretically identified candidates were not confirmed in the laboratory.^35^ Furthermore, quantitative experimental solubility data for PA6 and PET across solvents are limited, and polythermal (cloud/clear-point) measurements are typically carried out only up to the solvent’s boiling point (*T*_boil_), even though operation above the boiling temperature in pressurized vessels is easily realized in an industrial process. To the best of our knowledge, there is no publication so far that focuses specifically on PA6 and PET solubilities in different solvents. For the rational design of dissolution-precipitation processes for mixed plastic waste, we lack a standardized, above-*T*_boil_-capable, quantitative method to obtain polymer solubilities beyond subjective visual data.

Therefore, we developed a differential scanning calorimetry (DSC) method, which provides quantitative information for measuring temperature-dependent PA6 and PET solubilities in different solvents. We aim to answer the following research questions: Do decrystallization and precipitation temperatures measured using DSC correlate with the classical visual polythermal method? What added benefits compared to the visual method does DSC have for solvent selection? Can temperature-dependent selectivity windows determined by DSC be transferred to a practical PA6-PET separation?

In this article, by answering the research questions, we report the following novelties: a direct comparison of a DSC and visual polythermal method to measure polymer solubilities (Section 3.2) as well as PA6 and PET solubility correlations in nine organic solvents below and above the solvents’ boiling points (Section 3.3). Using DSC in Section 3.4, we identify solvents that are selective for PA6 but do not decrystallize PET in a solvent-based recycling process combining dissolution with temperature-induced precipitation. Lastly, applying DSC results in a proof-of-concept experiment, we separate PA6 and PET using methanol above its boiling point as the solvent (Section 3.5). The developed DSC method can easily be extended to measure the solubility of other polymers in solvents, thereby providing a consistent measurement framework in the development of solvent-based recycling processes.

## 2 Materials and Methods

Used polymers and solvents, as well as the experimental and analytical procedures applied, are described in this section.

### 2.1 Polymers and Solvents

Pure PA6 powder (PA6p, CAS RN 25038-54-4) with a mean particle size of *D*_50_ = 48 μm and pure PET powder (PETp, CAS RN 25038-59-9) with a mean particle size of *D*_50_ = 144 μm were purchased from Goodfellow (Cambridge, United Kingdom). Nylon PA6 low-warp filament with a diameter of 1.75 mm was bought from Spectrum Filament (Pęcice, Poland) and cut into 1-cm-long pieces (PA6g), and pure PET granules (PETg) with dimensions of roughly 2 × 2.2 × 2.7 mm were purchased from Goodfellow (Cambridge, UK).

HPLC grade methanol (MeOH, CAS RN 67-56-1) and ethanol (EtOH, CAS RN 64-17-5) with a purity of 99.9% were purchased from Merck (Darmstadt, Germany). Butyl lactate (BL, CAS RN 138-22-7) with a purity of 99% was purchased from Acros (Geel, Belgium). Diethyl oxalate (DEO, CAS RN 95-92-1) with a purity of 99% was purchased from Alfa Aesar (Haverhill, USA). Dimethyl sulfoxide (DMSO, CAS RN 67-68-5) with a purity of 99.5%, propylene glycol (PG, 1,2-propanediol, CAS RN 57-55-6) with a purity of 99%, ethylene glycol (EG, CAS RN 107-21-1) with a purity of 99.5%, and γ-valerolactone (GVL, CAS RN 108-29-2) with a purity of 99% were purchased from Sigma Aldrich (St. Louis, Missouri, USA). Propionic acid (PPA, CAS RN 79-09-4) with a purity greater than 99.7% was purchased from VWR Chemicals BDH (Poole, UK). Butyric acid (BA, CAS RN 107-92-6) with a purity greater than 99% was purchased from TCI (Tokyo, Japan). Milli-Q water (H_2_O, CAS RN 7732-18-5) with a resistivity of 18.2 MΩ·cm was prepared using a Milli Q IQ 7000 with a Millipak 0.22-μm filter.

All of the substances were used as received.

### 2.2 Visual Method to Determine Polymer Solubility

To determine the decrystallization temperature *T*_decr_ and the precipitation temperature *T*_prec_ of PA6 in a solvent, a fixed weighted amount of PA6p was suspended in a solvent together with a glass-coated stir bar in a 35 mL Pyrex vessel from CEM Corporation (Matthews, United States). The sample was gradually heated from 22 to 150 °C within 15 min under stirring (600 rpm) and then cooled to 22 °C within 10 min using a Discover 2.0 microwave synthesizer from CEM Corporation. Heating the system with microwaves compared to conventional convective heating does not change the thermodynamics of the phase transition.^44,45^ The microwave equipped with an optical camera allows for a visual inspection of the polymer particle decrystallization and precipitation in the solvent and tolerates pressures up to 20.7 bar, which was crucial due to the high vapor pressures of some solvents at 150 °C.

The temperature at which PA6p decrystallized and the solution cleared up (clear point) is noted as *T*_decr_, which can be found in the Supporting Information file S1, in ref 46. If PA6p did not decrystallize upon reaching 150 °C, the temperature was held for 10 min to overcome possible kinetic barriers. An incomplete PA6p decrystallization is also noted in S1. *T*_prec_ reports the temperature for the first observed clouding of the mixture (cloud point), indicating the initiation of the polymer particles’ precipitation.

### 2.3 DSC Method to Determine Polymer Solubility

We applied DSC to measure the decrystallization and precipitation temperatures of PA6p and PETp in various solvents. Since some of the evaluated solvents exert a high vapor pressure at the melting temperature of the polymer (*T*_melt_), a DSC3+ equipped with high-pressure stainless steel crucibles and gold-coated rupture discs from Mettler Toledo (Columbus, USA) was applied, which can withstand up to 300 °C and 100 bar. We assume the solvent vapor pressure to have a negligible effect on *T*_decr_ and *T*_prec_.^47^

Two thermal cycles were executed in the calorimetric studies to ensure a uniform thermal history of the polymer samples: samples were heated from 25 to 250 °C (PA6-containing samples) or 280 °C (PET-containing samples), cooled to −10 °C, heated again to 250 °C (PA6) or 280 °C (PET), and finally cooled to 25 °C with 20 K/min for the first heating and second cooling and 10 K/min for the first cooling and second heating (see Figure S1). The DSC chamber was consistently flushed with 30 mL N2/min. Some solvents were observed to decompose or depolymerize PETp at temperatures >200 °C, so the maximum temperature was lowered for the respective polymer–solvent combinations (for details, see the Supporting Information files S2, S3, and S4, in ref 46). We report *T*_prec_ as the peak during the first cooling and *T*_decr_ as the peak during the second heating. Further details of the developed DSC method can be found in the Supporting Information file S2, in ref 46.

### 2.4 Analytical Methods

Different analytical techniques were applied to study the characteristics of rPA6 and rPET in comparison to their virgin starting materials, PA6g and PETg.

#### 2.4.1 DSC for Melting Point Measurements

*T*_melt_ and the crystallization temperature of the polymer (*T*_crys_) of virgin PA6g and PETg as well as rPA6 and rPET were measured as in Section 2.3 but in the absence of solvents, as detailed in the Supporting Information file S2, in ref 46. The average of three sample measurements together with their sample standard deviation is reported. The method described here is typical for DSC analysis of PET in the literature.^34,48–50^

#### 2.4.2 XRD for Crystal Structure Determination

Powder X-ray diffractograms (XRD) were created by beaming K_α_ radiation from a Cu anode (λ = 1.54 Å) onto a sample and measuring the diffraction angle 2θ from 10 to 80° using a D2-Phaser diffractometer by Bruker Corporation (Ettlingen, Germany) at an operating voltage of 30 kV, a current of 10 mA, and a step size of 0.04, with a scan rate of 1/s. For comparable results, powdery rPA6, PA6p, and PETp were analyzed.

#### 2.4.3 SEC for MWD Determination

The molecular weight distribution (MWD) of PA6 and PET samples was measured as reported in our previous work^51^ by dissolving the polymer in hexafluoroisopropanol (HFIP), subjecting it to size exclusion chromatography (SEC, PSS PSG 100 Å, 7 μm, 8 × 300 mm and PSS PSG 1000 Å, 7 μm, 8 × 300 mm, 0.7 mL/min HFIP as eluent) and evaluating its MWD against a polymethyl methacrylate standard. Integration was carried out from 5 × 10^2^ to 2 × 10^6^ g/mol.

#### 2.4.4 GC for Extractables from rPA6 in Solvents

Gas chromatography (GC) was applied to the methanol supernatant after the dissolution–precipitation process to analyze whether PA6 degradation products or monomers were extracted from the polymer and did not precipitate. This was compared to extractables from virgin PA6g using boiling methanol at 65 °C (no decrystallization or dissolution). Using an Intuvo 9000 GC equipped with a HP-5MS UI column (30 m, 320 μm, 0.25 μm) and a flame ionization detector (FID) from Agilent (Santa Clara, USA), 1 μL of the sample was injected into the split injector at 250 °C with a split ratio of 50:1. The flow rate of the N_2_ carrier gas was 1.085 mL/min (constant flow mode). The column was held at 100 °C for 7 min before it was heated to 190 °C with a heating rate of 10 K/min. Then, 190 °C was held for 20 min before heating the column to 300 °C with a heating rate of 50 K/min and holding for 30 min to elute any impurities remaining on the column. The FID burning H_2_ and air operated at 320 °C and recorded currents at a sampling rate of 20 Hz. Agilent software version 3.3.65 was applied for the evaluation of the peak areas.

#### 2.4.5 Mastersizer for PSD Determination

The particle size distribution (PSD) of PA6p, PETp, and rPA6 was measured using a Mastersizer 3000 with Hydro EV as an accessory, purchased from Malvern Panalytical (Malvern, UK), detecting particles from 0.01 to 3000 μm. Particles or suspensions were introduced into the dispersion unit, stirring water at 2400 rpm. Through laser scattering of a red and blue laser in the detection cell, the particle size was calculated based on Mie scattering theory. From the resulting volume-based PSD, values like D10, D50, or D90 were calculated, indicating the particle diameter at which a specific percentage of the sample volume (10, 50, 90%) is accounted for. The particle size averages of three measurements are reported. Details of the applied standard operating procedure (SOP) are given in the Supporting Information file S6, in ref 46.

## 3 Results and Discussion

In Section 3.1, we discuss the applicability of the DSC method for polymer–solvent systems; in Section 3.2, we compare the visual and the DSC method. In Section 3.3, the temperature-dependent solubilities of PA6 in several solvents are shown, and in Section 3.4, the solvents which are selective for PA6 but do not decrystallize PET are discussed. Finally, in Section 3.5, we present the characterization of the recycled materials (rPA6 and rPET) and compare them to virgin materials (PA6g and PETg).

### 3.1 Representative DSC Curve of a Polymer in a Solvent

Both the decrystallization and chain disentanglement contribute importantly to the complete dissolution of polymer chains into a solvent.^52^ The decrystallization of a polymer in a solvent is an endothermic phase transition, and precipitation is an exothermic phase transition. Therefore, the DSC method is applicable as a simple method for temperature-dependent solubility studies for polymers. An endothermic calorimetric event in a mixture of polymer and solvent below *T*_melt_ can be attributed to the polymer decrystallization, while a lack of an endothermic event at *T*_melt_ demonstrates complete decrystallization of the polymer by the solvent. Likewise, if no exothermic event occurs during polymer-solution cooling at *T*_crys_ and an exothermic event is observed below *T*_crys_, the polymer remains fully decrystallized until the precipitation occurs at the observed exothermic event.^52^

A representative DSC curve of a polymer in a solvent is shown in Figure 2, in this case 36 wt % PA6p in methanol solution. The endothermic melting peak of pure PA6p at 212 °C is missing during the heating of the mixture (blue and red). Instead, endothermic peaks are observed at much lower temperatures during heating, indicating decrystallization. Also, the exothermic crystallization peak of pure PA6p at 171 °C is not visible during the cooling of the mixture (black and green). On the contrary, exothermic precipitation peaks are detected during cooling, indicating precipitation. This confirms our rationale in the paragraph above.

**Figure 2:**
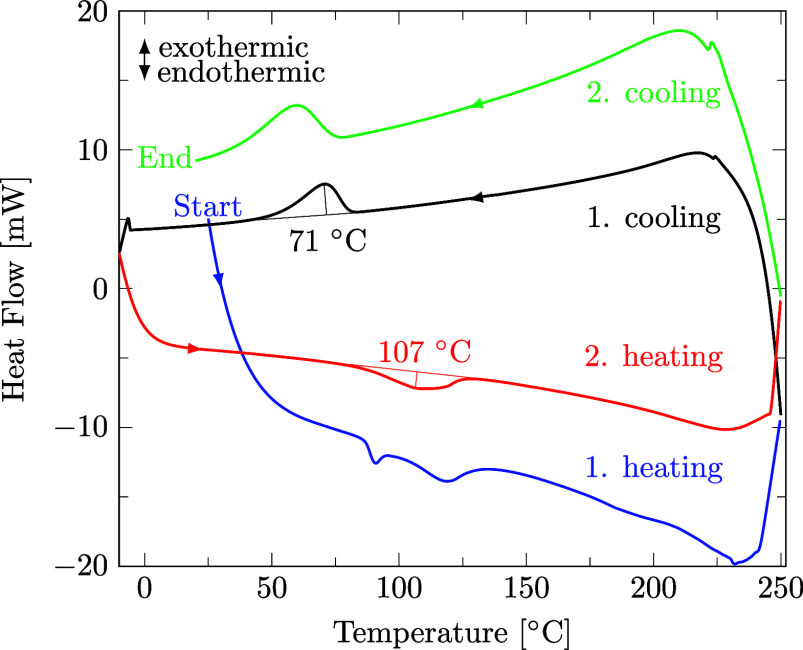
Representative DSC profile of PA6p in methanol: first heating (blue), first cooling (black), second heating (red), and second cooling (green). Endothermic and exothermic events indicate decrystallization and precipitation, respectively.

Figure 2 shows an example of the decrystallization peak during the second heating (red) being more reliable for integration than the decrystallization peak from the first heating (blue). The decrystallization peak from the first heating often changes appearance based on the mixing of the polymer and solvent in the crucible, as well as the crystallinity of the virgin material. Therefore, we evaluated the decrystallization peak of the second heating (red).

Furthermore, the sharper precipitation peak during the first cooling (black) was usually detected at higher temperatures compared to that during the second cooling (green). This shift is potentially caused by partial degradation of the polymer in the solvent at high temperatures, and therefore, the peak temperature of the first cooling (black) is evaluated to avoid reporting values for degraded polymers.

Note that the onset and offset of a thermic event are often 20 to 30 K apart with a peak between them. This implies that at the applied heating and cooling rate of 10 K/min, decrystallization or precipitation does not happen at one specific temperature but across a temperature range. We attribute this behavior to the dispersity of the polymer.

Lastly, Figure 2 shows no evaporation of the solvent methanol at its boiling point due to the closed vessel, although methanol becoming supercritical with peaks at ∼240 °C is observable. A similar phenomenon was also observed for other solvents. DSC signals of the pure polymer and pure solvent are included in S3 and S5.^46^

### 3.2 Comparability of the Visual and DSC Method

The first question to answer is whether the visual method for determining polymer solubility delivers the same results as the DSC method. Actually, the results of the two methods are very well comparable, as can be seen in Figure 3 for the three exemplary solvents: methanol, ethanol, and dimethyl sulfoxide. DSC raw data and the evaluation of the DSC and visual method results can be found in the Supporting Information files S1, S3, S4, and S5, in ref 46. S1 includes videos of PA6p decrystallization and precipitation in methanol and ethanol.

**Figure 3:**
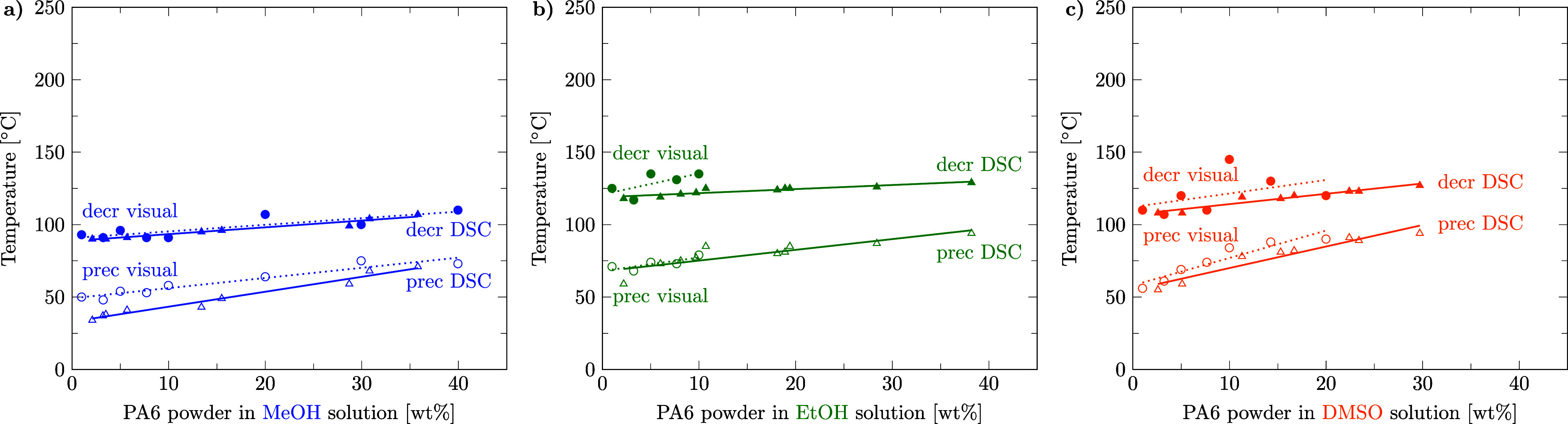
Comparison of the visual and DSC method. Decrystallization (decr, full symbols) and precipitation (prec, empty symbols) temperatures for PA6 powder (*D*_50_ = 48 μm) in a) methanol, b) ethanol, and c) dimethyl sulfoxide solution. Circles represent data points obtained from the visual method with dotted linear regression lines. Triangles represent data points from the DSC method with solid linear regression lines.

Methanol and ethanol becoming supercritical at 240 °C as well as the eutectic crystallization peak of DMSO-PA6 are visible in the DSC. For ethanol and dimethyl sulfoxide, it was not possible to obtain visual data points for high polymer concentrations due to viscosity limitations.

We observed a slight tendency that the visual method delivers higher temperatures for the decrystallization and precipitation of PA6 in methanol, ethanol, and dimethyl sulfoxide than the DSC method. This might have two reasons: First, for the visual method, the last decrystallized particle (clear point ≡ DSC offset) and the first precipitating particle (cloud point ≡ DSC onset) are visually observed, while the DSC peak temperature is reported. Second, although the heating and cooling rates are closely equal in both methods, a slightly higher cooling rate of the visual method may lead to a higher detected precipitation temperature compared to the DSC method. Further elaboration can be found in Section 3.4.

Compared with the visual method, the DSC method has several advantages: DSC works with opaque systems and is more objective and therefore reproducible because no clear point or cloud point has to be determined by the eye. DSC tolerates higher solvent vapor pressures, allowing for higher decrystallization temperature measurements, and requires only a few milligrams of solvent and solute compared to the several grams needed for the visual method. DSC is less time-consuming for the experimentalist when using an autosampler and delivers enthalpies of phase transitions, which are important for modeling of energy requirements. Two disadvantages of the DSC method compared to the visual method are the difficulty for the experimentalist to weigh in prescribed weight fractions and the absence of mixing in the crucible. In our view, the first disadvantage can be overcome by interpolating between multiple data points, and the second disadvantage is negligible due to the small sample volume where we observed homogenized polymer and solvent in the DSC crucible.

### 3.3 Temperature-Dependent Solubilities of PA6

We selected nine potential solvents and antisolvents for PA6 from the literature^15,35,53^ for quantitative evaluation. Applying DSC, we report the temperature-dependent solubilities of PA6 in nine solvents, even above the boiling points of the solvents. The decrystallization temperature (*T*_decr_) and precipitation temperature (*T*_prec_) as key metrics are evaluated in this section. We observed that, in general, a strong solvent lowers the temperature at which the solute changes phases (also called melting point depression or crystallization temperature depression), but not the energy needed for this, as the enthalpy depends primarily on the crystalline fraction of the polymer and not the polymer concentration.^52^ We observed a nearly linear dependence of *T*_decr_ for PA6 as a function of the polymer concentration in solution measured with the DSC method (see Figure 4). Individual data points were omitted in Figure 4 for clarity but can be found in the Supporting Information files S3 and S5, in ref 46. It is well visible that weak solvents for PA6 like diethyl oxalate and butyl lactate do not significantly lower the phase change temperature of PA6, while strong PA6 solvents like acids (propionic acid and butyric acid) significantly lower the phase transition temperature of PA6 compared to the melting temperature of pure PA6p (*T*_melt_) indicated by the dashed line (205.1 ± 1.4 °C). We observed that for some solvents, *T*_decr_ increases with increasing PA6 concentration, while for other solvents, *T*_decr_ remains constant over a large concentration range. An increase can be explained by reduced contact between solvent and polymer, lowering cohesion as well as reduced vapor and osmotic pressure at higher polymer concentrations.^52^

**Figure 4:**
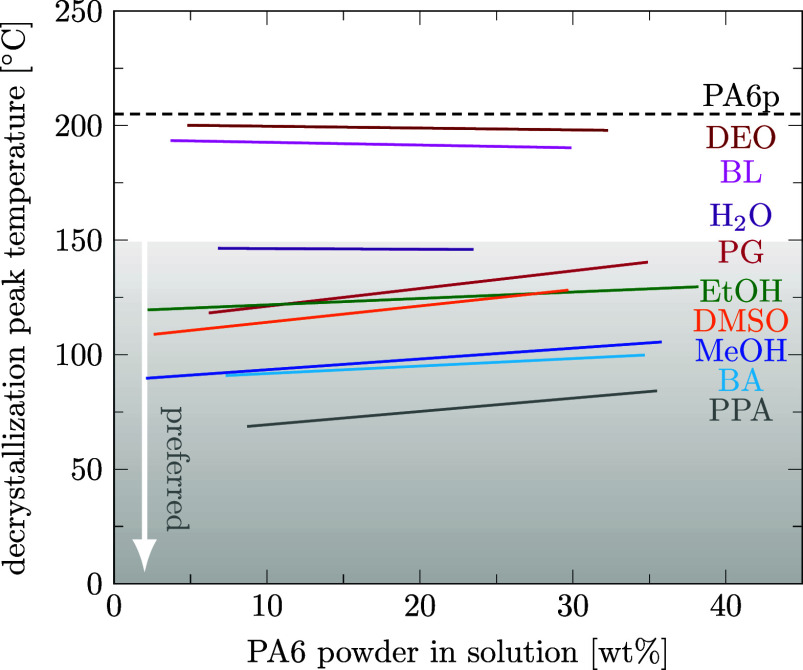
Linear regression of the decrystallization temperatures measured by DSC with PA6 powder (*D*_50_ = 48 μm) as a function of polymer weight fraction in solution. Solvents are diethyl oxalate (DEO), butyl lactate (BL), water (H_2_O), propylene glycol (PG), ethanol (EtOH), dimethyl sulfoxide (DMSO), methanol (MeOH), butyric acid (BA), and propionic acid (PPA). The melting temperature of pure PA6 powder is marked with a dashed line for comparison purposes. To avoid PA6 decomposition, a decrystallization temperature below 150 °C is preferred.

Since we observed PA6 degradation at temperatures above 150 °C, we prefer to look for solvents in which *T*_decr_ remains below this temperature, as indicated by the gray shading in Figure 4.

As shown for *T*_decr_, we observe a closely linear dependence of *T*_prec_ as a function of the polymer amount in solution. Figure 5 shows linear regression lines of *T*_prec_ for PA6 powder in different solvents measured by DSC, with detailed data points given in the Supporting Information files S3 and S5, in ref 46. In line with the literature,^47,52,54,55^ we have found that for a fixed composition, decrystallization (*T*_decr_) occurs at significantly higher temperatures than precipitation (*T*_prec_) for all investigated solvent–polymer combinations. Similar to Figure 4, weak solvents for PA6, such as diethyl oxalate and butyl lactate, do not clearly show a lower phase transition temperature compared to the crystallization temperature of pure PA6p (*T*_crys_) as indicated by the dashed line (163.7 ± 2.1 °C). Strong solvents for PA6, such as acids (propionic acid and butyric acid), lower *T*_prec_ of PA6 substantially. In the case of propionic acid, the polymer did not even precipitate during the measurement; hence, data for propionic acid as a solvent are not shown in Figure 5. Also similar to Figure 4, *T*_prec_ increases with increasing PA6 concentration for some solvents, while for other solvents, *T*_prec_ remains constant over a wide concentration range.

**Figure 5:**
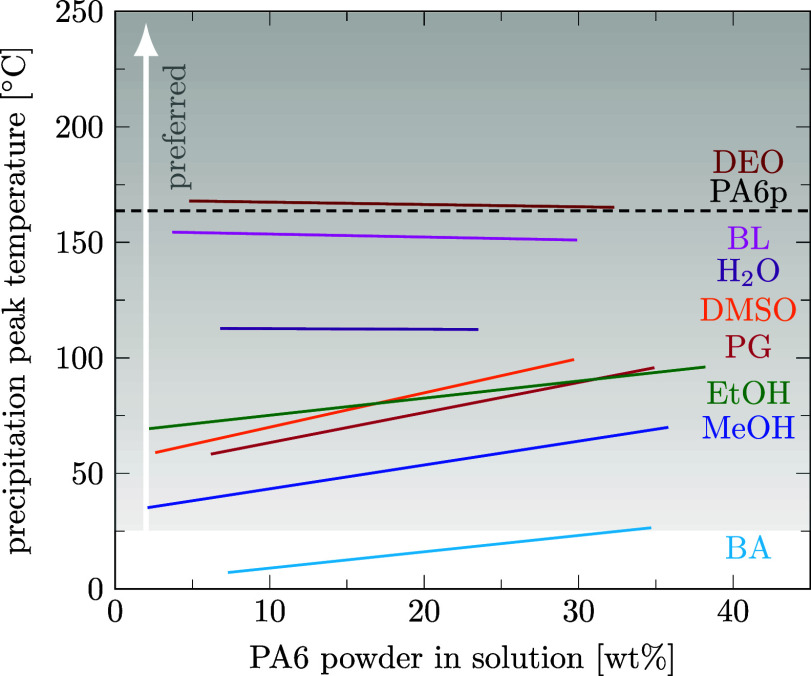
Linear regression lines for the precipitation temperatures measured by DSC for PA6 powder (*D*_50_ = 48 μm) as a function of polymer weight fractions in solution. Solvents are diethyl oxalate (DEO), butyl lactate (BL), water (H_2_O), dimethyl sulfoxide (DMSO), propylene glycol (PG), ethanol (EtOH), methanol (MeOH), and butyric acid (BA). The crystallization temperature of pure PA6 powder is marked with a dashed line for comparison purposes. Solvents with precipitation temperatures for PA6 above room temperature are preferred to avoid extensive cooling.

To avoid the need for a refrigeration machine for temperature-induced precipitation, precipitation is preferred at or above room temperature (indicated with gray shading in Figure 5). Since we observed a low *T*_decr_ of solvents to correspond to an even lower *T*_prec_, we did not investigate very strong solvents for PA6, such as formic acid, phenols, or calcium chloride-alcohol mixtures.

Concluding, preferred solvents for PA6 are methanol, ethanol, propylene glycol, dimethyl sulfoxide, and water, which show significant lowering of *T*_decr_ compared to *T*_melt_ of pure PA6p and also exhibit a sufficiently high *T*_prec_.

### 3.4 Solvent Selectivity and Comparison with Literature

Preferred solvents for PA6 could also be strong solvents for PET. With regard to the design of the recycling process for mixed polymers, we aim at finding a solvent able to decrystallize only one of the two polymers contained in the mixture. Therefore, it is crucial to know the temperatures at which the second polymer is decrystallized and precipitated to be able to choose a selective solvent. This section is dedicated to the question if preferred solvents for PA6 also decrystallize PET and if our data are in agreement with literature data. For this purpose, as for PA6 (Section 3.3), the solubility of PET was measured in different solvents using DSC. Details of data points and regression lines reported in Figures 6–12 can be found in the Supporting Information files S3, S4, and S5, in ref 46.

**Figure 6:**
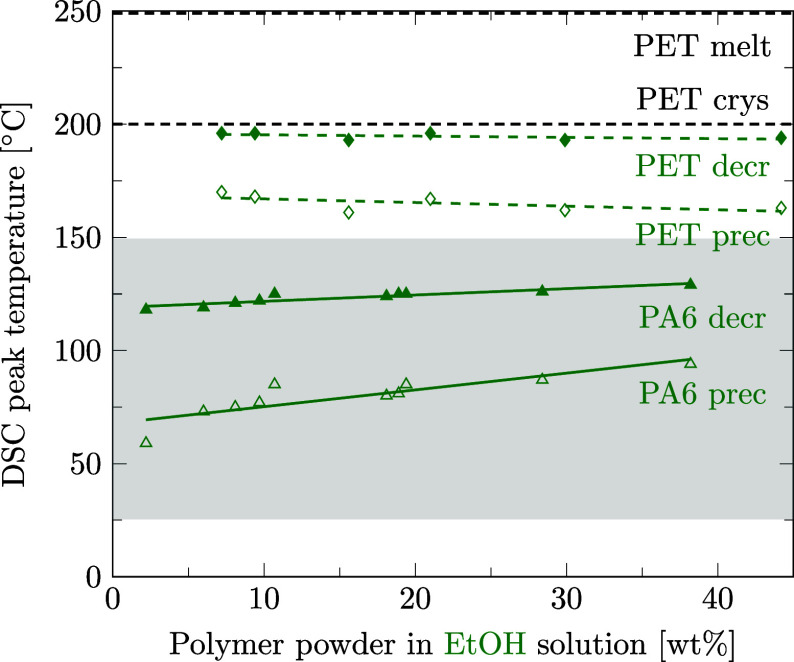
Decrystallization (decr, full symbols) and precipitation (prec, empty symbols) temperatures for PA6 (triangles) and PET (diamonds) powder in ethanol solution measured by DSC. Linear regression results are given for data points of PA6 (solid lines) and PET (dashed lines). Melting and crystallization temperatures of pure PET are marked with dashed black lines for comparison.

Figure 6 shows that ethanol is a solvent for PET since it lowers PET’s phase transition temperature during decrystallization as well as precipitation. However, in the temperature range of 25 to 150 °C, where PA6 is decrystallized and precipitated in ethanol, PET remains crystalline and could even be precipitated. The same applies to propylene glycol (Figure 7), methanol (Figure 8), and water (Figure 9).

**Figure 7:**
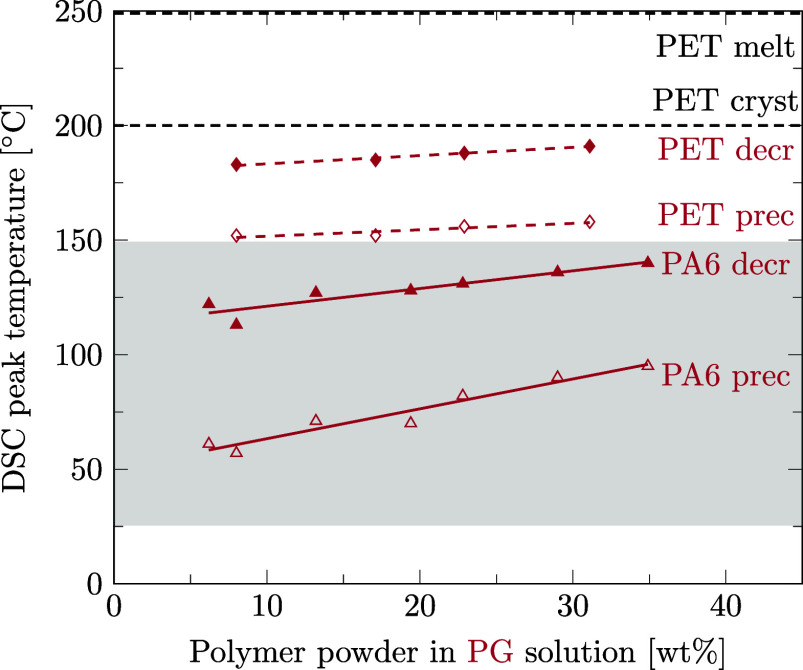
Decrystallization (decr, full symbols) and precipitation (prec, empty symbols) temperatures for PA6 (triangles) and PET (diamonds) powder in a propylene glycol solution measured by DSC. Linear regression results are given for data points of PA6 (solid lines) and PET (dashed lines). Melting and crystallization temperatures of pure PET are marked as dashed black lines for comparison.

**Figure 8:**
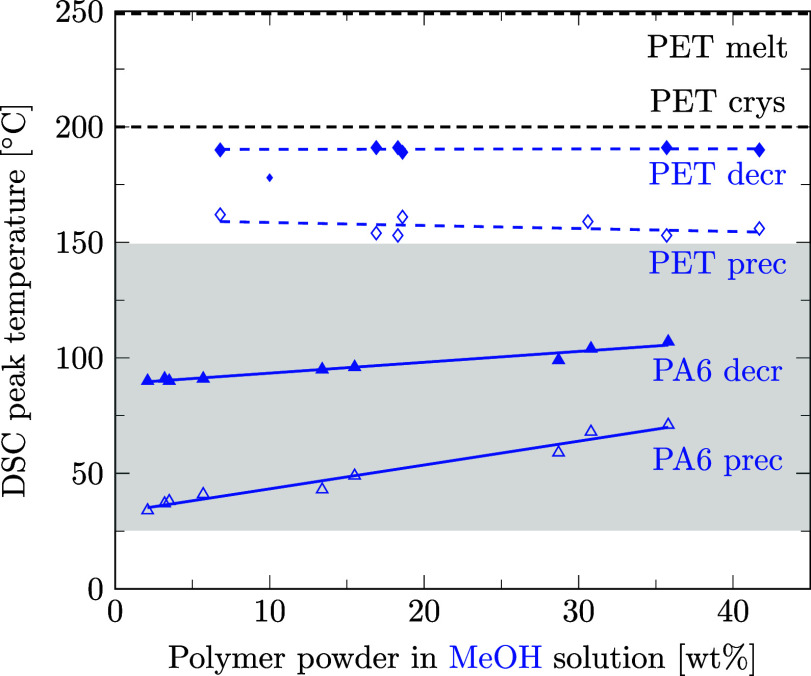
Decrystallization (decr, full symbols) and precipitation (prec, empty symbols) temperatures for PA6 (triangles) and PET (diamonds) powder in methanol solution measured by DSC. Linear regression results are given for data points of PA6 (solid lines) and PET (dashed lines). Melting and crystallization temperatures of pure PET are marked as dashed black lines for comparison. Literature data as a small marker.^48^

**Figure 9:**
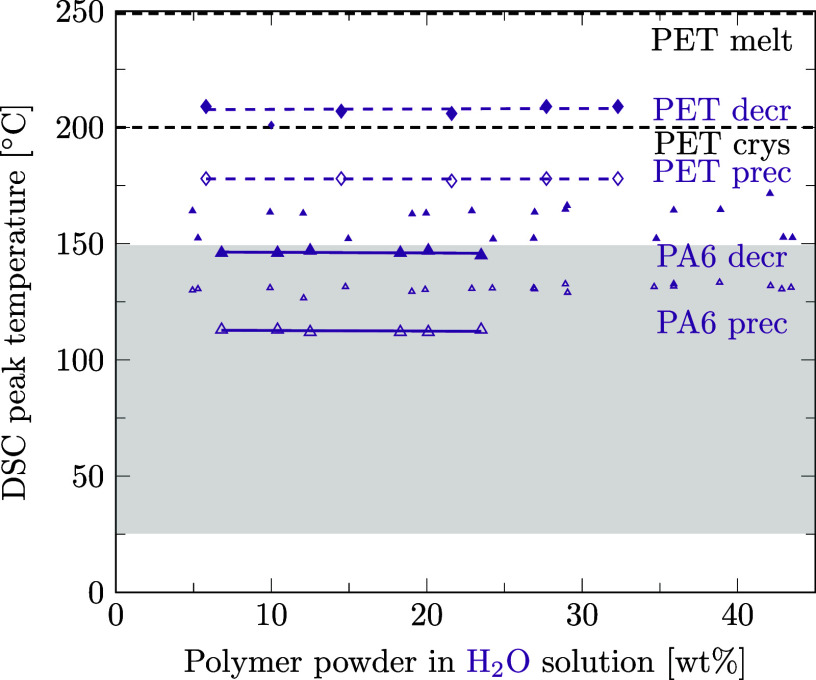
Decrystallization (decr, full symbols) and precipitation (prec, empty symbols) temperatures for PA6 (triangles) and PET (diamonds) powder in water solution measured by DSC. Linear regression results are given for data points of PA6 (solid lines) and PET (dashed lines). Melting and crystallization temperatures of pure PET are marked as dashed black lines for comparison. Literature data as small markers.^47,48,54,55^

In the few instances where literature data are available, they are inserted into Figures 8–12 with small markers. In general, there is good agreement between our data and the literature data.

For PA6 solubility, one data set was found^47,54,55^ in which the phase transition temperature of PA6 in water solution remains constant for concentrations below 60 wt % PA6 (Figure 9). This trend matches well with those of our measurements. It is well known that a lower molecular weight of the polymer leads to a lower phase transition temperature—with or without a solvent—but the measurement method of the phase transition temperature is even more decisive for the obtained result: while we report DSC peak temperatures in our study, the literature data set^47,54,55^ is based on offset temperatures for decrystallization and onset temperatures for precipitation. Therefore, systematically higher *T*_decr_ and *T*_prec_ values are reported in the literature than in the present study. In line with our DSC results (Figure 10), Papaspyrides and Kartalis (2000)^56^ report the dissolution of PA6 in dimethyl sulfoxide at temperatures between 110 and 130 °C, employing 125 °C in their solvent-based recycling process.

**Figure 10:**
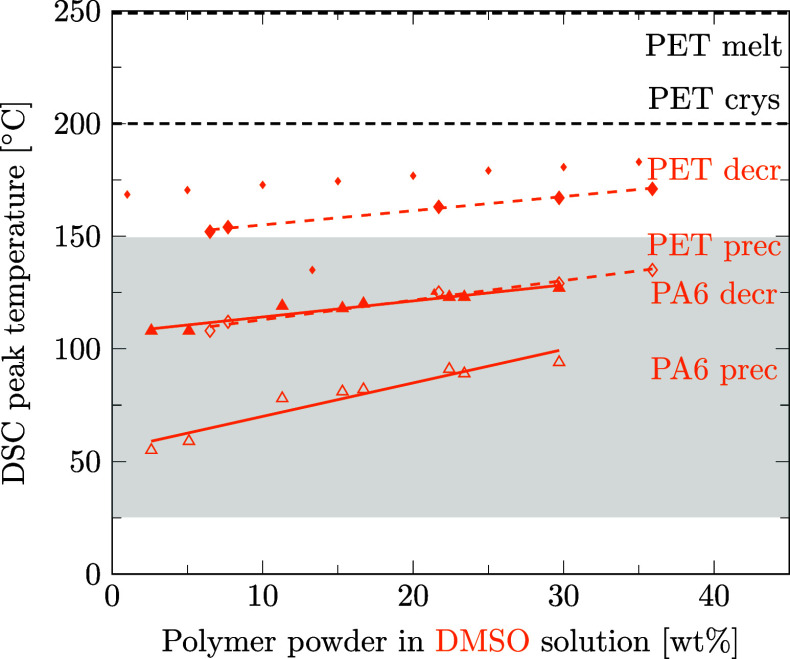
Decrystallization (decr, full symbols) and precipitation (prec, empty symbols) temperatures for PA6 (triangles) and PET (diamonds) powder in dimethyl sulfoxide solution measured by DSC. Linear regression results are given for data points of PA6 (solid lines) and PET (dashed lines). Melting and crystallization temperatures of pure PET are marked as dashed black lines for comparison. Literature data as small markers.^35,49,56^

Regarding PET solubilities in different solvents, there are a few more literature references than for PA6, although they are limited to *T*_decr_ and do not report *T*_prec_. It is worth noting that the literature data on PET is not always consistent. For PET in dimethyl sulfoxide, Schlüter et al. (2024)^49^ report higher phase transition temperatures than Zhou et al. (2023),^35^ whereas we measured the corresponding values in between (Figure 10). Also, for PET in γ-valerolactone (Figure 11)^49,57,58^ and ethylene glycol (Figure 12),^48,49^ our solubility measurements lie between the values reported in literature.

**Figure 11:**
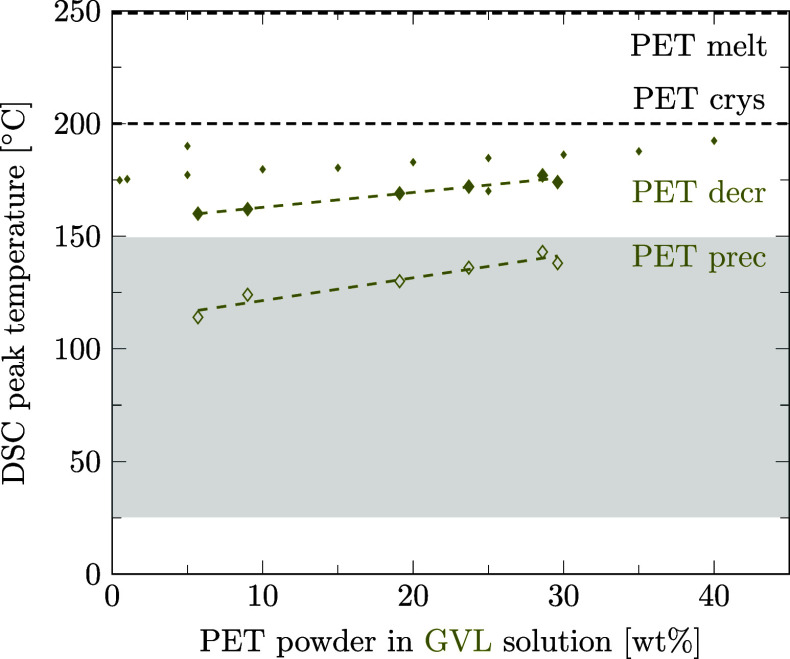
Decrystallization (decr, full symbols) and precipitation (prec, empty symbols) temperatures for PA6 (triangles) and PET (diamonds) powder in γ-valerolactone solution measured by DSC. Linear regression results are given for data points of PA6 (solid lines) and PET (dashed lines). Melting and crystallization temperatures of pure PET are marked as dashed black lines for comparison. Literature data as small markers.^49,57,58^

**Figure 12:**
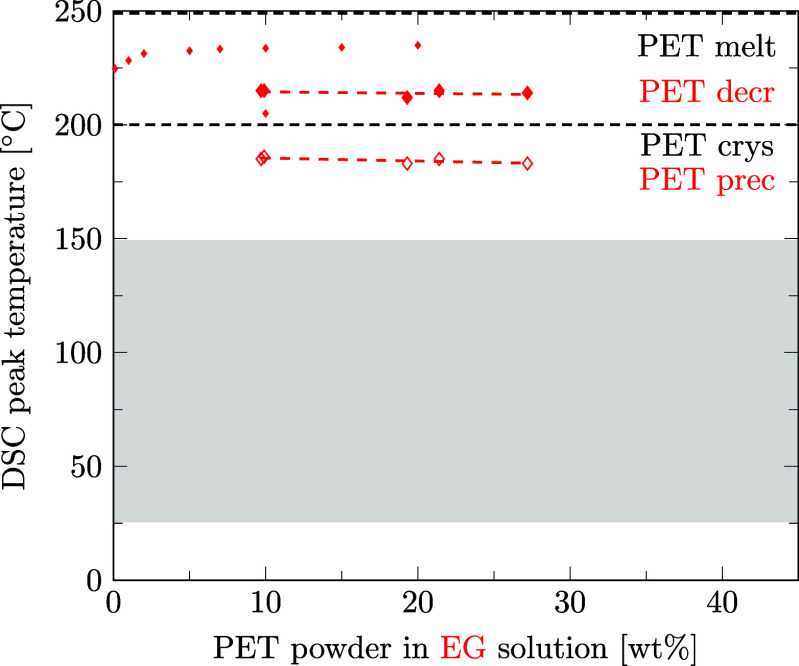
Decrystallization (decr, full symbols) and precipitation (prec, empty symbols) temperatures for PA6 (triangles) and PET (diamonds) powder in ethylene glycol solution measured by DSC. Linear regression results are given for data points of PA6 (solid lines) and PET (dashed lines). Melting and crystallization temperatures of pure PET are marked as dashed black lines for comparison. Literature data as small markers.^48,49^

As for PA6, the reported phase transition temperature of PET in a solvent depends primarily on the measurement method and barely on the minor differences in the molecular weight of the polymer. Schlüter et al. use a PET with a comparable molecular weight and melting point to our PET and report the clear point (≡ DSC offset) temperature, applying a polythermal method instead of the peak temperature for decrystallization of PET in dimethyl sulfoxide, γ-valerolactone, and ethylene glycol. This leads Schlüter et al. to systematically report values 10–15 K higher than those obtained in our study. With the same reasoning, Jaime-Azuara et al. (2024),^48^ who assess the onset temperature, systematically report lower values for *T*_decr_ of PET in methanol, water, and ethylene glycol compared to us, who report the peak temperature. Chen et al. (2021)^57^ observe the clear point of PET decrystallization in γ-valerolactone at a temperature closely equal to our reported DSC-peak values and lower than the values reported by Schlüter et al. possibly due to a significantly lower molecular weight of PET used by Chen et al. compared to PET used by us or Schlüter et al. Lastly, Zhou et al. measure clear point data for PET in dimethyl sulfoxide, reporting a much lower *T*_decr_ compared to data from us or Schlüter et al. This might be attributable to a significantly lower PET molecular weight, but neither molecular weight nor melting temperatures of pure PET are reported by Zhou et al. Thus, together with polymer solubilities, the exact evaluation method (onset, peak, or offset) and the molecular weight of the polymer should be reported in further polymer solubility studies.

Summarizing, for ethanol, propylene glycol, methanol, water, and dimethyl sulfoxide, sufficiently selective decrystallization can be achieved by controlling the temperature such that PA6 is decrystallized, but PET remains in the solid phase. Our DSC-based measurement results correspond well with the existing literature data.

### 3.5 Proof-of-Concept Experiment for Solvent-Based Recycling of PA6 and PET with Methanol

A proof-of-concept experiment was carried out using methanol in a solvent-based recycling process to selectively dissolve PA6 from a mixture of coarse particles of PA6g and PETg. We chose methanol to demonstrate the applicability of DSC results with a representative solvent featuring *T*_decr_ for PA6 above the solvent’s boiling point and because methanol has so far been scarcely investigated as a solvent for PA6 in the literature. Furthermore, in comparison with the other solvents investigated in this study, methanol is the best solvent regarding parameters like *T*_decr_ and *T*_prec_ for PA6 and the difference *T*_decr_ PET – *T*_decr_ PA6, as shown in the Supporting Information file S10, Table S1, in ref 46. Additionally, methanol is neither reported to degrade PA6 nor PET at *T*_decr_ for PA6, and it is biosourcable and also biodegradable. The potential of methanol as a selective solvent was also recognized in a patent,^59^ claiming methanol dissolves PA but not PP, rubbers, jute, PU, polyvinyl chloride, polymethyl methacrylate, wool, and PET.

Specifically, to obtain rPA and rPET samples for comparison with virgin polymer, 0.29 g of PA6g and 0.29 g of PETg were placed together into a 35 mL Pyrex vessel (CEM Corporation, Matthews, United States) with a glass-coated stir bar. 2.71 g of methanol was added to obtain concentrations of 10 wt % of each polymer in the solution. The maximal applicable polymer concentration was constrained due to viscosity limitations, namely 10 wt % polymer in solution in accordance with the literature.^13,33,60–65^ The mixture was heated up in the microwave synthesizer to *T*_decr_ = 109 °C and kept at this temperature for 10 min to ensure complete dissolution of PA6. *T*_decr_ was chosen 15 K above the linearly regressed value for the DSC peak (*T*_decr_ for 10 wt % PA6 in methanol solution; see results in Section 3.2) because the DSC offset of the decrystallization was often observed 10–15 K above the peak. *T*_prec_ was set to 28 °C, corresponding to the complete precipitation of PA6. Again, since the DSC offset of the precipitation was often observed 10–15 K below the peak, *T*_prec_ was chosen 15 K below the linearly regressed DSC peak (*T*_prec_ for 10 wt % PA6 in methanol solution). After precipitation of rPA as a fine powder, both polymers were resuspended in an additional 3 g of methanol and filtered through a sieve with a mesh size of 1.1–1.2 mm. The rPET granules remaining on the sieve were washed with another 30 g of methanol. The collected rPA in methanol was filtered using a vacuum filtration setup with Whatman grade glass fiber filter paper (GF 92) procured from Cytiva. Drying of both polymers was done in a drying chamber at *T*_dry_ = 75 °C for 2 h since we found this sufficient to evaporate all methanol without the need for a vacuum. The mass balance of the polymers was calculated to detect possible decomposition, insufficient precipitation, or solvent residues in the polymers.

During the selective dissolution experiment in the microwave synthesizer, methanol was observed to start boiling at 65 °C. All PA6g had dissolved at *T*_decr_ = 109 °C under the established autonomous system pressure of 4.2 bar gauge. Simultaneously, PETg neither decrystallized nor stuck and visually appeared untouched in the clear PA6-methanol solution. During cooling, the system pressure dropped expectedly to atmospheric pressure at 65 °C, and PA6 was observed to start precipitating at 48 °C as a fine powder. These observations closely match the results for *T*_decr_ and *T*_prec_ of pure PA6p in methanol measured by DSC. Thus, apparently, the presence of PET in the mixture does not cause significant changes in PA6 behavior in methanol solution.

#### 3.5.1 Characterization of Recycled Polymers

rPA6 and rPET were subjected to a few analytical methods, as described in Section 2.4. The results are summarized in Table 1.

**Table 1:** Characterization of Virgin and Recycled Polymers from Dissolution and Precipitation in Methanol

Analysis	Virgin PA6g	rPA6	Virgin PETg	rPET
Crystal structure [2θ]	20.2 and 23.8° [PA6p]	20.2 and 23.8°	16.4, 17.6, 22.8, 25.9° [PETp]	-
*T*_melt_ ± STD [°C]	192.2 ± 0.3	195.3 ± 0.7	250.3 ± 0.2	252.5 ± 0.1
*T*_crys_ ± STD [°C]	145.1 ± 0.4	147.4 ± 1.1	214.4 ± 0.8	214.2 ± 0.5
MWD (*M*_*n*_ and *M*_*w*_) [10^3^ g/mol]	19.9 and 59.2	26.3 and 63.2	22.0 and 57.4	20.8 and 51.5
Monomer in solvent	0.18 wt %^a^	0.55 wt %	-	-
Particle size	1.75 × 10 mm	*D*_10_ = 12 μm, *D*_50_ = 47 μm, *D*_90_ = 130 μm	2 × 2.2 × 2.7 mm	

a 10 wt % PA6g treated in boiling methanol.

First, the crystal structure of rPA6 measured by XRD (Figure 13) shows the same 2θ angles as purchased virgin PA6p. These angles correspond well to diffraction angles of 20.2° and 23.5° as reported for PA6 in the literature,^29,66^ indicating that not only the same polymer but also a comparable semicrystallinity had formed during precipitation, which is common for virgin PA6p. Additionally, none of the characteristic PETp angles are observed in rPA6, indicating the desired separation of the two polymers. Details of the XRD results can be found in the Supporting Information file S7, in ref 46.

**Figure 13:**
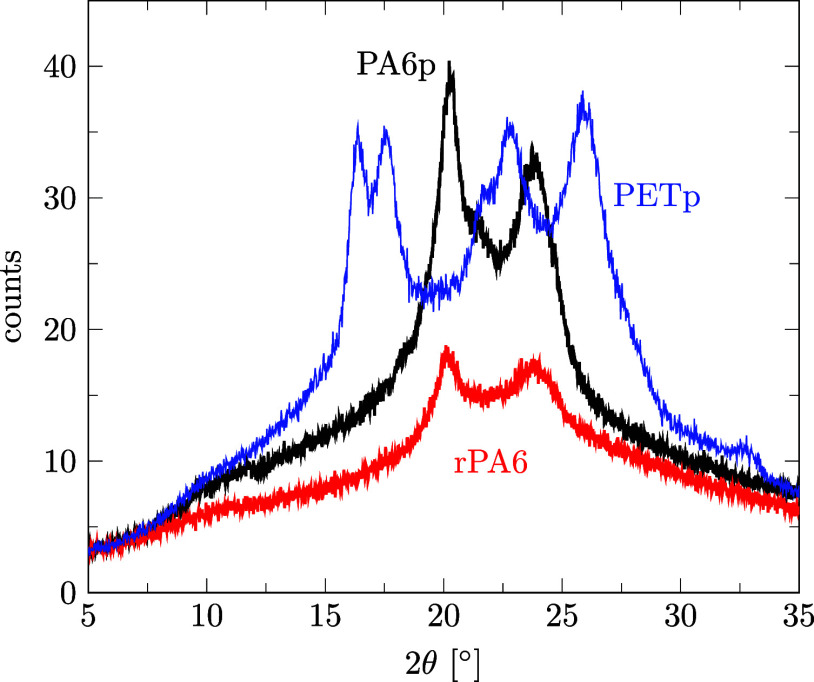
XRD spectra of rPA6 compared to that of virgin PA6p and PETp.

Second, during DSC analysis (Figure 14), no methanol evaporation out of rPA or rPET was observed; therefore, we assume all methanol to have evaporated from the samples during drying. There are no PET peaks for melting or crystallization observed in the PA6 samples nor vice versa, so the two polymers were completely separated. Additionally, the average of *T*_melt_ and *T*_crys_ of rPA6 and rPET across three samples, including the sample standard deviation (STD), is closely equal to those of virgin PA6g and PETg, which is a strong indication that the recovered polymers are free of degradation, cross-contamination, and solvent traces. Details of the DSC analyses can be found in the Supporting Information files S3, S4, and S5, in ref 46.

**Figure 14:**
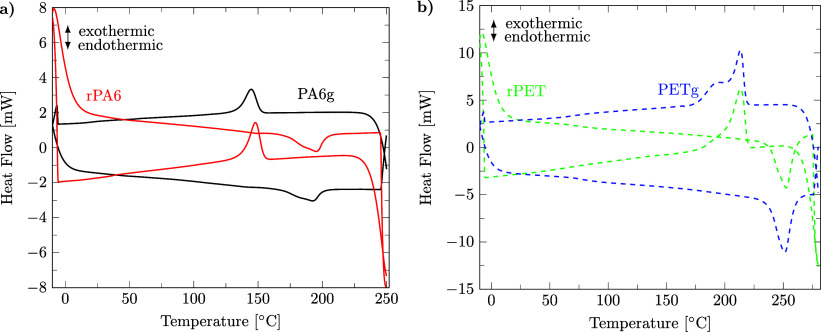
DSC analysis shows virgin and recycled polymers after selective dissolution and precipitation: a) PA6 and b) PET.

Third, the MWD of recovered and virgin polymers remained closely comparable (Figure 15). For rPA6 compared to PA6g, the number average molecular weight (*M*_*n*_) increased by 32% and the mass average molecular weight (*M*_*w*_) increased by 7%. This increase results from the fewer low molecular weight mono- and oligomers in rPA6 compared to PA6g, which likely remained in the solution (see GC results below) and were not reprecipitated. As PET was not dissolved by methanol, low molecular weight fragments (∼750 g/mol) of PETg remained in rPET, which were not extracted from the polymer. For rPET compared to PETg, *M*_*n*_ decreased by 5%, and *M*_*w*_ decreased by 10%. These changes are most often deemed acceptable in the literature, given a 10% accuracy of the SEC analysis, as discussed in our previous work.^51^ The dispersity of PA6 and PET remained between 2 and 3. Detailed MWD evaluations, including SEC raw data for PA6p and PETp, can be found in the Supporting Information file S8, in ref 46.

**Figure 15:**
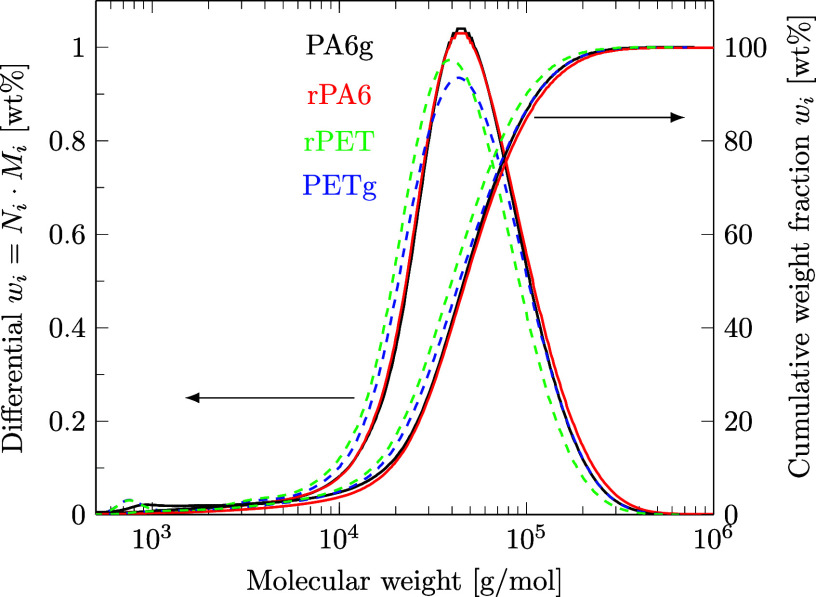
Molecular weight distributions of rPA and rPET compared to those of virgin PA6g and PETg determined by SEC.

Fourth, the extraction of PA6 fragments into the solvent during the recycling process was analyzed. GC analyses of the recovered solvent showed that 0.55 wt % of the PA6g was extracted as the monomer caprolactam into methanol. For comparison, when PA6g (10 wt % of the solution) was treated in boiling methanol for 10 min (no decrystallization or dissolution), only 0.18 wt % of the PA6g mass got extracted as caprolactam into methanol. The gas chromatograms, as well as a calibration curve, can be found in the Supporting Information file S9, in ref 46. It is unclear whether the three times higher caprolactam yield in methanol after dissolution compared to treatment in boiling methanol resulted solely from caprolactam that was originally enclosed in the PA6 and released during the dissolution or whether the polymer degraded, yielding caprolactam as a product. SEC results do not suggest a degradation of PA6 into low molecular weight mono- or oligomers but rather their loss to the solvent. Even if rPA6 were slightly degraded into caprolactam, a loss of 0.55 wt % would be bearable for the recycling process.

Fifth, the mass balance closure as the ratio of recovered polymer mass to input polymer mass is nearly 100% (101% for PET and 92% for PA6). The slight deviation from the ideal value might be caused by PA6 decomposition, insufficient precipitation of PA6, or PA6 losses in the vessel or the filter. Since neither do the other analyses support the degradation of PA6 nor are there significantly high traces of polymer in the solvent, we suspect losses in the experimental equipment. It can be assumed that the polymer mass balance closure will get very close to 100% in larger than lab-scale volumes > 1L as reported by Papaspyrides and Kartalis (2000).^56^

Sixth, rPA6 precipitates as fine particles (Figure 16) that are very similar in size to the commercially obtained PA6p (*D*_10_ = 19, *D*_50_ = 48, *D*_90_ = 87 μm), with a slightly broader PSD. Details of the particle size distribution measurements can be found in the Supporting Information file S6, in ref 46. Small PA6 particles precipitating from methanol were described elsewhere too^67^ and are highlighted to ease postprocessing in comparison to melts due to easier additivation and enhanced desolventation.^58^

**Figure 16:**
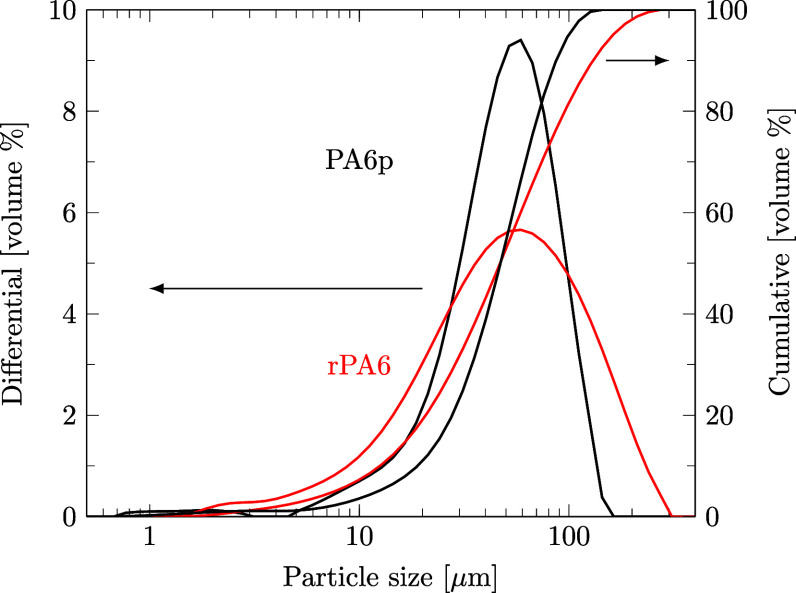
Particle size distribution (PSD) of PA6p and precipitated rPA particles obtained after selective dissolution and precipitation.

Consequently, rPA6 and rPET could be separated using a solvent-based selective dissolution and precipitation process with methanol as the solvent and remained mostly unaltered, as proven by different analytical techniques.

## 4 Conclusions

Literature on temperature-dependent polymer solubilities, especially for PA6 and PET, is scarce and sometimes inconsistent because different metrics are evaluated with different experimental methods. In this work, we have presented a standardized DSC method that provides quantitative data on polymer decrystallization and precipitation in various solvents. In comparison to the visual polythermal method, DSC allows for more objective and reproducible measurements and saves time for the experimentalist. Moreover, DSC can operate under higher pressures, expanding the search space in polymer decrystallization studies to temperatures above the solvent’s normal boiling points. Applying the DSC method, it is possible to determine not only the clear point (DSC heating offset) and cloud point (DSC cooling onset) but also the temperatures at which the polymer starts decrystallizing (DSC heating onset) and at which all of the polymer has precipitated from solution (DSC cooling offset). This enables strict and objective criteria to include or exclude potential solvents during a screening, which is of relevance to process studies. Even while requiring only a few milligrams in sample size, DSC delivers comparable results to other experimental methods that operate on the gram scale.

We filled a prevalent literature gap by reporting temperature-dependent PA6 solubilities in diethyl oxalate, butyl lactate, water, propylene glycol, ethanol, dimethyl sulfoxide, methanol, butyric acid, and propionic acid, as well as temperature-dependent PET solubilities in water, propylene glycol, ethanol, dimethyl sulfoxide, methanol, γ-valerolactone, and ethylene glycol in a consistent measurement framework, showcasing the melting point depression being larger for strong solvents and smaller for weak solvents. Our solubility results align well with literature values. We found methanol, ethanol, propylene glycol, dimethyl sulfoxide, and water showing sufficient decrystallization selectivity for a solvent-based recycling process with temperature-induced precipitation to separate PA6 from PET.

In a proof-of-concept experiment, we demonstrated that methanol above its normal boiling point can be applied to selectively dissolve PA6 from PET and that the two polymers are not degraded in the process. The low-boiling solvent has the potential to reduce the heating requirements for polymer drying in the recycling process, thereby enhancing its economical and ecological performance.

## Data Availability

Supporting data for this article are available on EDMOND at 10.17617/3.MEFXPY under a CC BY 4.0 License.
